# Individual diversity between interdependent networks promotes the evolution of cooperation by means of mixed coupling

**DOI:** 10.1038/s41598-019-47013-x

**Published:** 2019-08-01

**Authors:** Sicheng Liu, Lin Zhang, Baokui Wang

**Affiliations:** 1 School of Automation Science and Electrical Engineering, Beihang University, Beijing, 100191 China; 2 Engineering Research Center of Complex Product Advanced Manufacturing Systems, Ministry of Education, Beijing, 100191 China; 3 Joint Exercises and Training Center, Joint Operations College, National Defense University, Beijing, 100091 China

**Keywords:** Coevolution, Computer science

## Abstract

Along with the rapid development of network-based information technology, such as cloud computing, big data, the IoT, and so on, human society has stepped into a new era of complex networks. People’s life and production activities depend more and more on various complex networks to ensure security and reliability. The complex interrelationships between human and nature establish a link to explain the cooperation of individual behaviour, especially for individual diversity. However, existing researches mostly ignore the influence of individual diversity on networks involved in individual behaviour to strategy selection. Therefore, it needs further research on how to consider both individual diversity and independent networks in the evolution of cooperative behaviour. To address this issue, we extend a simple game model into the interdependent networks through the mixed coupling (i.e., utility and probability) in this work. Also, we divide the kinds of strategic behaviour of a player in one layer concerning individual diversity. Moreover, there exists an optimal region of mixed coupling between networks such that cooperation can be promoted. Finally, experimental results can open the path to understanding the emergence and maintenance of cooperation within various interconnected and interrelated real-world systems newly.

## Introduction

The cooperation has been widely recognized as the existence in the evolution of species, ranging from multicellular organisms to human societies. It is a question that the emergence and maintenance of cooperation between selfish or unrelated individuals in the context of Darwinian evolution have plagued the scientific community^1–3^. The evolutionary game theory that can provide a unifying mathematical framework^4^ to think about the collective action in real-world systems, such as the educational distribution, social welfare, environmental pollution and so on, answers such a question. Besides, the public goods game (PGG) as one of the most typical representative, is generally used for the problem of distribution between the individual and the collective^5^. Then, defectors can get the same benefit of the cooperators without the cost. As a result, it would be expected to result in the tragedy of the commons. Except for main five kinds of well-known rules^6^ to be devoted to resolving this social dilemma, several novel mechanisms, such as diversity^5,7^, social influence^8–10^, reputation^11,12^, co-evolution^13–16^, punishment^17^, reward^18,19^, rubust^20^ and so on, have been sprung up to support the evolution of cooperation.

The last few years have seen sustained progress in the science and technology, especially for well-known complex networks theory which attracts more and more scholars from the interdisciplinary research to investigate the optimal representation of the underlying complex systems and mechanisms through harnessing the power of the Internet of Things and Big Data. Examples arrest to the fact that seemingly irrelevant changes in one network can have catastrophic and very many unexpected consequences in another network^21^. The key to understanding these phenomena is multi-layer networks^22–24^, and in particular, happened on how individual behaviour available on one network influence the evolution of cooperation on the other network. However, although evolutionary game on interdependent networks become increasingly popular to draw scholarly attention^25,26^, the focus still mires on stage referring to various evolutionary dynamics on networks^27–32^, interdependent structured populations^33,34^ and single connection. There are two kinds of connection style on networks. The difference is whether the connection style has a physical connection or not. There is no physical connection to be referred to correlation of utilities^9,35–41^, coupling of interaction^32,42–44^, learning graphs^45^ and resources reallocation^46^. To compare our model with existing research^16,42,45,47,48^, we convey the three crucial points. The mixed coupling exists extensively in the real world, however, either utility function^47^ (belong to non-physical connection) or probabilistic interconnection^42^ (belong to physical connection) only consider one aspect of connection style. Moreover, the selection of individual behaviour is static but dynamic and real-time, which cannot mobilize to the maximum extent individual’s subjective initiative to cooperate. Additionally, individual diversity is reflected in the unique nature of individual selection, while the scaling factor^48^ considered limiting the other network to adopt a strategy. Furthermore, there is few research on mixed coupling affiliated to individual interactions, which represents the consideration of every possible angle. Therefore, it makes more sense to study the existence of mixed coupling. Consider a cloud manufacturing system, where three layers account for three participants and their interdependencies (CMfg operators, resource providers and resource demanders). Resource providers involved in production can be paid access to resources in the cloud resource pool to meet the individual needs of resource demanders under the operation of CMfg operators. CMfg operators obtain payoffs from resource providers to publish idle resources and resource demanders to access the resources in the cloud resource pool. Except for above interaction relationship (can be regarded as one kind of coupling), these participants also need to satisfy restricting condition (can be regarded as another kind of coupling), such as precedence constraints and allocation rules. Within each layer, participants compete for the different maximum benefit (minimizing the makespan, minimizing the total workload and minimizing the total energy consumption), which reflect the characteristics of the individual diversity. Thus, a natural question arises as to whether the mixed coupling of interdependency in a real system is a relevant factor for the emergence and survival of cooperative behaviour.

In this paper, we extend the scope of evolutionary games on interdependent networks by introducing individual diversity through the mixed coupling (i.e., utility and probability) in this work. To shed light on the interdependence between networks, we use the mixed coupling to be depicted abstractly. Note that probability *p* = 0 both networks are entirely independent of one another, while probability *p* = 1 they can be viewed as an integral whole which is fully connected internally. However, the interdependence between networks does strictly obey the binomial distribution of probability 0 < *p* < 1. For simplicity sake, we employ the two-layered lattices for both networks A and B. Moreover, individuals on both networks can play with all its neighbours, including the neighbour from the other layer. While in the strategic transfer process, players of both networks can learn their nearest neighbours including the possible one via an external link between networks, simultaneously dividing the kinds of strategic behaviour of a player in one layer concerning the individual diversity. Interestingly, we also show that there, in fact, exists simultaneously an optimal region of mixed coupling between networks for the cooperation to be promoted. A vast plethora of results validates the point that individual diversity between interdependent networks through mixed coupling can significantly promote the evolution of cooperation.

The remainder of this paper is organised as follows. In Section 2, we first describe the public goods game model considering interdependency between networks. Then, Section 3 is dedicated to the presentation of numerical results. Finally, the concluding remarks are brought in Section 4.

## Model

**Figure 1:**
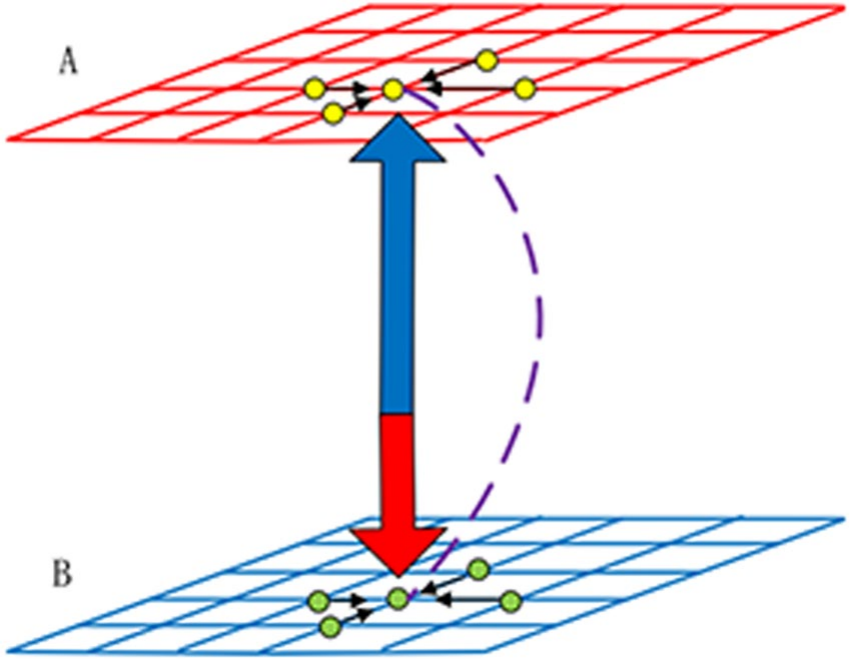
Schematic presentation of the model. Depending to the scheme, the coupling between the upper network A (red) and lower network B (blue) is not only by the utility but also by the probabilistic interconnection (purple). Here, we only discuss the case of one-to-one correspondence between both networks.

For ease of comparison with previous studies^35,42^, we will also use the most common model of public goods game. The evolutionary game is staged on two *L* × *L* disjoint square lattices with periodic boundary conditions and von-Neumman neighbourhoods, where each player is thus surrounded by its *k* = 4 nearest neighbours. Likewise, a fraction *p* of players on network A is randomly selected and allowed to form an external link with a corresponding player on network B, as seen in Fig. 1 for schematic representation. It should be noted, however, that interdependency between different networks is one-to-one.

Initially, an equal percentage of strategies (cooperators or defectors) is randomly distributed between networks. A player *x* on network A acquires payoffs *P*_*x*_ by playing the PGGs with all its neighbours. Namely, cooperators contribute an amount of *c* = 1 to the public goods pool, while defectors contribute nothing. The total contribution is subsequently multiplied by an enhancement factor *r* (*r* > 1), reflecting the synergetic effects of cooperative group, and then, it is equally shared between all involving members of the group irrespective of their strategies. The payoff ${P}_{x^{\prime} }$ of player *x*′ on network B can be obtained in the same way as *P*_*x*_. Here, the player interacting with its neighbours can also occupy sites on the other network.

To quantitatively take the utility of each player on interdependent networks into account at the same time, we will consider the following way

1 $$\{\begin{array}{rcl}{U}_{x} & = & {P}_{x}+{\alpha }_{1}{P}_{x^{\prime} }\\ {U}_{x^{\prime} } & = & {P}_{x^{\prime} }+{\alpha }_{2}{P}_{x}\end{array}$$

where (*α*_1_, *α*_2_) is utility coupling coefficient set 0 < *α*_1_, *α*_2_ < 1 determining the strength of an external link for each player’s utility *U*_*x*_ (${U}_{x^{\prime} }$) incorporated by the *P*_*x*_ and ${P}_{x^{\prime} }$ on their host lattices. For (*α*_1_ = 0, *α*_2_ = 0), it will result in the fact that *U*_*x*_ (${U}_{x^{\prime} }$) is entirely determined by the *P*_*x*_ (${P}_{x^{\prime} }$) identically as if considered on an isolated network. While for (*α*_1_ = 1, *α*_2_ = 1), *P*_*x*_ will be seen as the equal degree as ${P}_{x^{\prime} }$, which means that the individual mutual coupling will not be translated into the difference. Based on the former research^35^, where they have studied the general impact of the value of *α* between networks, therefore we use (*α*_1_ = 0.5, *α*_2_ = 0.5) here, which shows that the individual utility will be mainly decided by its payoff and moderately related to the corresponding partner on the other network. It is important to emphasize that the network mode is symmetric, the roles are exchanged for *α* > 0.5.

Then, player *x* randomly chooses among its nearest neighbours *y* and imitates its strategy *S*_*y*_ with a probability according to the imitation dynamics. Here, the strategy transfer between networks can be carried out.

2 $$W({S}_{y}\to {S}_{x})={w}_{y}\frac{1}{1+\exp [({U}_{x}-{U}_{y})/K]}$$

where *K* quantifies the uncertainty in the strategic transfer^49^. Since the selected value of *K* does not qualitatively affect the evolutionary outcomes, we will adopt *K* > 0.1 according to the pioneer contributor^50^. The scaling factor *w*_*y*_ represents the individual behaviour diversity and depicts the impact of the strategy to transfer of player *y* in different layers.

3 $${w}_{y}=\{\begin{array}{ll}\mathrm{1,} & if\,y\in A\\ 1-\beta \frac{{N}_{x}}{G}, & if\,y\in B\end{array}$$

where *y* belonging to network A (i.e., *y* ∈ A) is regarded as an influential player, who can convince its neighbours to adopt its strategy with a higher payoff when compared to network B (i.e., *y* ∈ B). *N*_*x*_ is the number of players in that group *G* = *k* +1  centred on the corresponding one *x*’ on network B, which adopts the same strategy as player *x* on network A. *β* is the multiplicative factor 0 < *β* < 1 to avoid frozen states. To be consistent with the previous study^48^, we apply *β* = 0.5 in this work. Hence, all players on separate networks will be divided into two types of players regarding strategic diffusion, to reflect the difference in individual behaviour within the real-world populations as far as possible.

The linear system size was varied from *L* = 100 to 300 in order to avoid finite-size effects. We verify that the presented results do not qualitatively change for reasonable variations. Also, simulations of the model were performed using the synchronous update, where each player on networks had a chance to interact in their respective neighbourhood, and then all sites are updated simultaneously through competition with a randomly chosen neighbour once on average during a Monte Carlo step (MCS). If not stated before, the equilibration is required up to 2 × 10^4^ steps and then sampled by another 2 × 10^3^ steps. These final results were averaged over 50 different independent runs to further improve accuracy.

## Results

**Figure 2:**
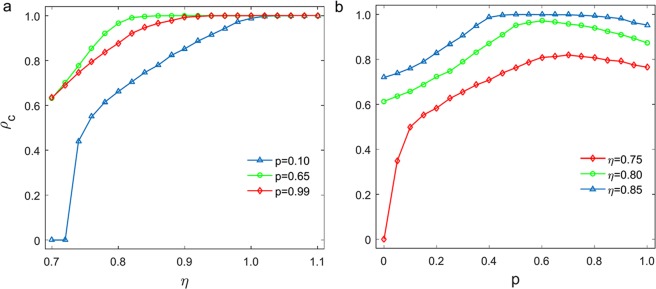
The evolution of cooperation on two interdependent networks as a function of *η* with different values of *p* (left panel) and as a function of *p* with different values of *η* (right panel).

To evaluate the impact of individual diversity on the evolution of cooperation, we firstly present in Fig. 2 the frequency of cooperators *ρ*_*c*_ for PGG played in interdependent networks. Figure 2a shows the frequency of cooperators *ρ*_*c*_ as a function of the renormalised enhancement factor *η* = *r*/(*k* + 1 + *p*) with different values of probabilities *p*(0 ≤ *p* ≤ 1), while Fig. 2b shows the frequency of cooperators *ρ*_*c*_ as a function of *p* with different values of factor *η*. We observe that strategy transfer between networks promotes faster cooperation when *p* = 0.65. Moreover, there also exists an optimal region of *p* maximising the fraction of cooperators for each fixed *η*, which is in line with the previous results^42^. The only difference is that the optimal region of *p* moves to the more extensive range of values, not intermediate value. As a result, the cooperation between interdependent network will evolve to an optimal state characterised by individual diversity.

**Figure 3:**
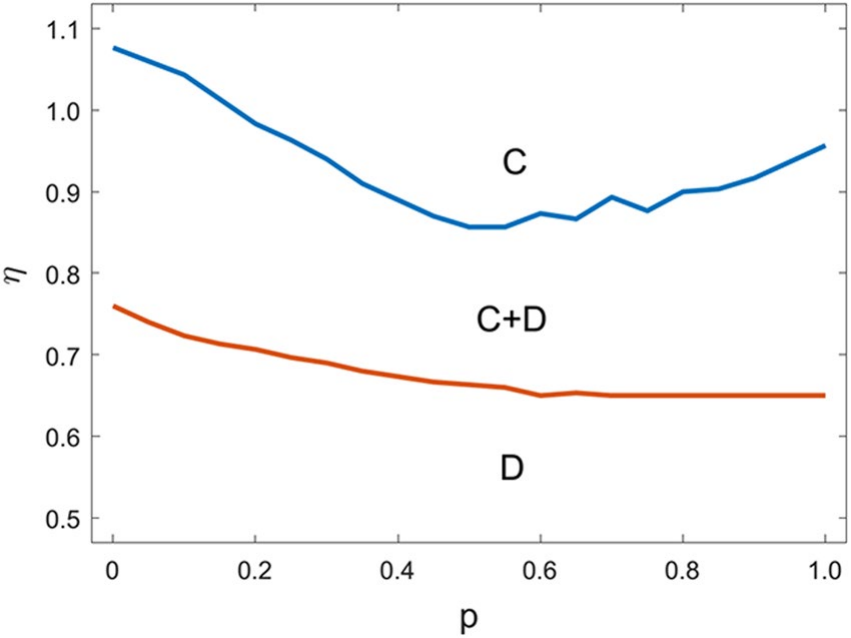
The complete *p* − *η* phase diagram for PGG on the interdependent networks. The upper (lower) boundary is the extinction threshold of cooperators (defectors) correspondingly.

Figure 3 shows the result of the full *p* − *η* phase diagram for the evolution of cooperation on the interdependent networks. We observe that the fraction of cooperators and defectors descend with increasing *p* and tend to be stable. Somewhat differently, the fraction of cooperators is with small fluctuations. Hence, the range of coexistence of cooperators and defectors is delimited by two threshold values for *η*. Below the low extinction threshold, cooperators vanish. While above high extinction threshold, defectors are doomed. Meanwhile, near the low extinction threshold, cooperator pairs tend to annihilate and disappear. Whereas near the high extinction threshold, single defector and defector pairs cannot survive in a sea of cooperators. That is, the strongest coupling effect occurs in the extinction thresholds for cooperators and defectors. Thus, individual diversity promotes the formation of a strong coupling effect on the evolution of cooperation to some extent.

**Figure 4:**
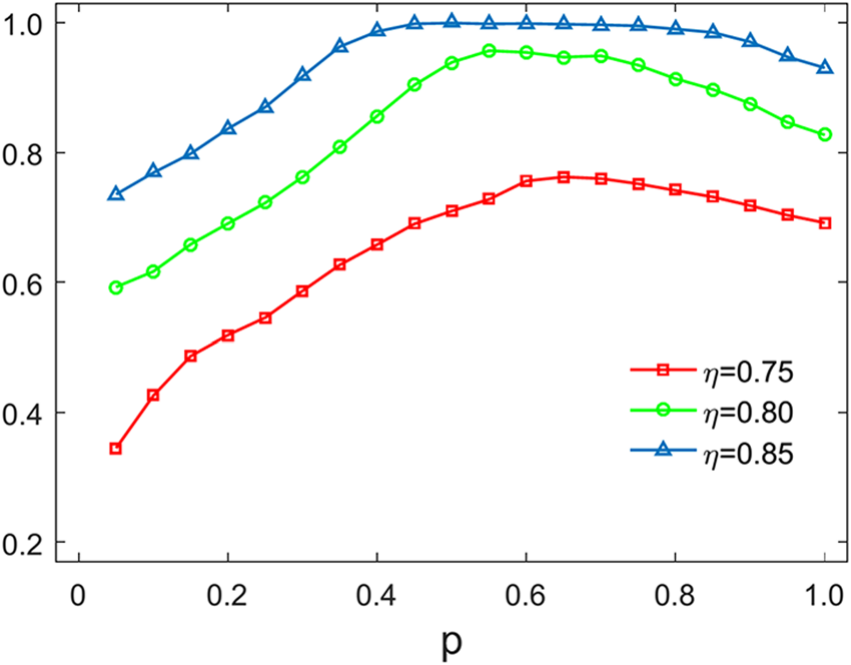
The frequencies of CC links between interdependent networks as a function of *p* with different *η*.

Figure 4 illustrates the frequencies of conditional cooperation (CC) strategies of two corresponding individuals between networks at equilibrium with different *η* regardless of whether there is a connection between them or not. One can see from Fig. 4 that the frequencies of CC strategies between networks closely resemble the evolution of cooperation as shown in Fig. 2b for the corresponding *η*. This phenomenon indicates that the individual diversity between interdependent networks refers to a coupling effect, which can significantly influence on the evolution of cooperation. To elaborate the relationship between CC strategies and interdependent networks, we use the simplified correlation coefficient.

4 $${r}_{c}=({\rho }_{cc}-{\rho }_{c}^{2})/({\rho }_{c}-{\rho }_{c}^{2})$$

where *ρ*_*cc*_ denotes the fraction of CC strategies of two corresponding individuals between networks. When *ρ*_*c*_ satisfies the range of 0 to 1, *r*_*c*_ becomes greater than 0 for different *η*. So the cooperative behaviour across interdependent networks can be promoted through the individual diversity, which can be of certain signs to the evolution of cooperation.

**Figure 5:**
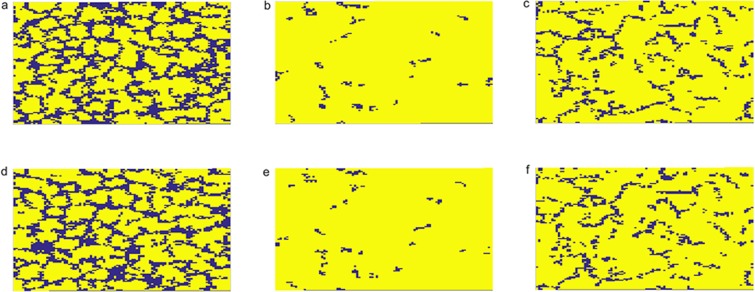
Snapshots of the typical distribution of cooperators (yellow) and defectors (blue) on two interdependent networks at *p* = 0.1, 0.65, 0.99 from left to right. Panels (a–c) correspond to results obtained on network A, while panels (d–f) correspond to results obtained on network B. Parameter values are: *η* = 0.8 and *β* = 0.5.

For an intuitive understanding of the positive effects of individual diversity between interdependent networks on the evolution of cooperation, it is useful to look at characteristic snapshots in such a stationary state of given values as shown in Fig. 5. With the effect of individual diversity, the distribution of cooperators and defectors are fundamentally similar in the two networks for different *p* above. Not so surprisingly, however, a relatively tiny fraction of defectors in the middle row of Fig. 5 (panels b and e) can survive by forming large, compact clusters, thus reducing exploitation by cooperators, which gives rise to the fact that cooperators dominate perfectly on both networks. By contrast, defectors form small filament-like clusters in the third row of Fig. 5 (panels c and f), which cooperators have an advantage over others to maintain cooperation between interdependent networks. In the first row of Fig. 5 (panels a and d), in fact, both do the coexist, approximately the one on the evolution of an isolated lattice, respectively. As a consequence, strong coupling effect made by the individual diversity brings out significantly constraint, in turn, affects the persistence of cooperation.

**Figure 6:**
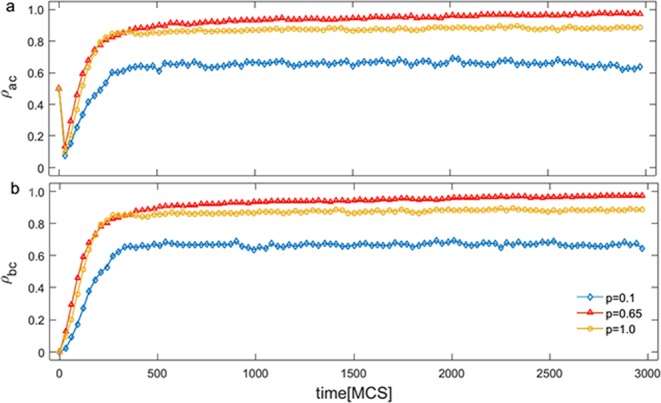
Time evolution of the frequency of cooperators in simulations on networks (**a**) (*ρ*_*ac*_) and (**b**) (*ρ*_*bc*_) with different initial conditions for *η* = 0.8. Initially, network (**a**) is randomly distributed with the equal proportion of the two strategies while network (**b**) is held by one strategy (defectors).

Figure 6 shows the time evolution of the frequency of cooperators in simulations with different initial conditions, which serve well to highlight two strategic invasion processes across networks. It helps to show that the frequency of cooperators on network A monotonously increases to level off after declining sharply over time for different *p* shown in Fig. 6a. While that on network B is a first monotone increase, then tends to a relatively steady state approximation shown in Fig. 6b. That is, no doubt, a very remarkable fact, but the frequency of cooperators between networks are closer to the level of almost cooperation for optimal *p*. It is conspicuous, the strongest coupling effect made by the individual diversity appears at p = 0.65 not always intermediate value, which significantly reflects the different time evolutions.

## Discussion

In summary, we have introduced the simple game model considering heterogeneity into the interdependent networks using the mixed coupling (i.e., utility and probability). Meanwhile, we divide the kinds of strategic behaviour of a player in one layer concerning individual diversity. This effect demonstrates that the synergy between network reciprocity and the spontaneous emergence of correlated behaviour on the two networks can come into being.

Besides, we have shown that there, in fact, exists simultaneously an optimal region of mixed coupling between networks for the cooperation to be promoted. Moreover, the cooperation level on networks can be greatly enhanced with increasing *η*. Utilizing calculating the frequencies of CC strategies and the correlation coefficient of CC strategies, the frequencies of CC strategies are precisely similar to the evolution of cooperation on both networks as a function of *p* with different *η*. It implies that the evolution of cooperation on networks is perfectly correlated with the frequency of CC strategies for the corresponding *η*. Moreover, snapshots of the typical distribution of cooperation have shown that the network interdependence fundamentally changes the formation of clusters on both networks. Importantly, there is a reason to believe the individual diversity through the probabilistic interconnection and utility functions enhances the coupling effect between interdependent networks indeed. Therefore, the way of constructing between networks is just like a bridge interacting with each other, which represents a status of “you have me, and I in you”.

Too much interdependence is not practical either - there must also be sufficient independence for the individual networks to remain functional if the evolution of cooperation in the other network goes wrong. It can be proved the fact that Space X launch Falcon Heavy rocket, which has rocket’s Fregat strap-on boosters as few as possible while guaranteeing high reliability. Moreover, it is also worth mentioning that the present model has difficulty in applying directly to a realistic situation because of simple model establishing, but it does not impede to capture the essence of some real life. Since the investigation of interdependent networks is a promising research topic, especially for the evolutionary games that can help to provide the more comprehensive understanding how cooperative behaviour among selfish individuals in sizable groups evolves to emerge, revealing the internal mechanisms, even to maintain real-world systems ranging from natural, biological, engineering ones to human societies. We hope that it can be exploited effectively to resolve social dilemmas as to inspire further studies, such as, heterogeneity, co-evolution of the interdependent way and strategy updating diversity, which will enrich the context of spatial reciprocity.
